# A global compendium of human dengue virus occurrence

**DOI:** 10.1038/sdata.2014.4

**Published:** 2014-05-27

**Authors:** Jane P Messina, Oliver J Brady, David M Pigott, John S Brownstein, Anne G Hoen, Simon I Hay

**Affiliations:** 1 Spatial Ecology and Epidemiology Group, Department of Zoology, University of Oxford, South Parks Road, Oxford, OX1 3PS, UK; 2 Department of Pediatrics, Harvard Medical School and Children’s Hospital Informatics Program, Boston Children’s Hospital, Boston, Massachusetts 02115, USA; 3 Department of Community and Family Medicine, Geisel School of Medicine, Dartmouth College, Hanover, New Hampshire 03755, USA; 4 Fogarty International Center, National Institutes of Health, Bethesda, Maryland 20892, USA

## Abstract

A global geographic database of human dengue virus occurrence was produced to generate a global risk map and associated burden estimates^1^. Herein we present the database, which comprises occurrence data linked to point or polygon locations, derived from peer-reviewed literature and case reports as well as informal online sources. Entries date from 1960 to 2012. We describe all data collection processes in full, as well as geo-positioning, database management and quality-control procedures. This is the most comprehensive database of confirmed human dengue infection to-date, consisting of 8,309 geo-positioned occurrences in total.

## Background & Summary

The quality of reporting of dengue virus infection is inconsistent by country and by region and is often biased by difficulties in diagnosis, limited resources for diagnostic testing and the varying reporting capacities of national health systems. Furthermore, active surveillance of dengue infection, which can often be asymptomatic, is rare, so it is difficult to gauge the limits and intensity of transmission in a consistent manner across the world using standard reported case data. In order to address these biases, this database focuses on occurrence rather than incidence of dengue virus infection which allows it to inform global risk models more accurately. The final database described here consists of records of known occurrences of dengue virus infection globally, each identified by its unique geographical location and the year in which it occurred between 1960 and 2012. Sources of information include published literature, case reports and informal online sources, described in detail in our methods section.

With this database, it is possible to model the probability of occurrence of dengue transmission with a high degree of spatial resolution, for example as in Bhatt *et al.*^1^, the first project to make use of it in its final form. Bhatt *et al.* derived the global probability of occurrence of dengue over long-term average conditions at a 5 km×5 km resolution using the database described here as well as a suite of environmental covariates described in the supplementary information to that paper. This final map was also used to support a project examining risk for dengue during the 2014 World Cup in Brazil^2^ and the database was also updated to contain type-specific information and re-summarised by province in a paper by Messina *et al.*^3^ which examines the spread of each of the four dengue virus types. Because the locations of dengue occurrence are recorded at the finest level of detail possible (i.e., points when the exact location was known or else polygons for administrative units when this was the best information available), the database may be used for dengue mapping and modelling at any spatial resolution required, with the only limiting factor being the resolution of the modelling covariates or the specific aims of the user.

It should be noted that we did not discriminate studies or reports based upon the clinical outcome of the dengue infections they reported; this information was not consistently reported across all of the varied sources and we aimed to be comprehensive in our inclusion of all known locations of dengue infection. Thus, while we included asymptomatic infections reported in the few existing prospective population-based seroepidemiological studies (i.e., cohort studies, which are the only type of study capable of detecting and measuring this type of infection), they were not distinguished from occurrences of the disease derived from symptomatic hospital-reported cases.

All data collection processes are described in full here, as well as the geo-positioning, quality-control and database-management procedures. The database’s construction from many different sources of information makes it the best currently available standardised data available on global dengue transmission. The result is the most comprehensive database of confirmed human dengue infection to-date, consisting of 8,309 geo-positioned occurrences in total worldwide. The database is not necessarily confined to use for global analyses, as it contains enough detailed information for many parts of the world to carry out modelling at a regional or even sub-national scale, with regions and countries specified in addition to the specific location of every record in order to facilitate sub-setting of the database for the user’s needs.

## Methods

### Data collection

The description of these methods is modified and expanded from the methods previously included in the Supplementary Information of Bhatt *et al.*^1^ PubMed (http://www.ncbi.nlm.nih.gov/entrez/query.fcgi) was searched using the term ‘dengue’ for the years 1960 to 2012. The Medical Subject Headings (MeSH) term technology used in the PubMed citation archive ensured all pseudonyms were automatically included (http://www.nlm.nih.gov/mesh) in the searches. The same process was repeated for ISI Web of Science (http://wok.mimas.ac.uk) and ProMED (http://www.promedmail.org). The searches were last updated on 10th October 2012. No language restrictions were placed on these searches; however, only those citations with a full title and abstract were retrieved. This resulted in a collection of 5,876 references, of which 2,883 unique articles were identified as potentially containing useable location data. The full texts were obtained for 2,838 of these (98.4%) and the information from 1,655 articles was ultimately included in our database. In-house language skills allowed processing of all English, French, Portuguese and Spanish articles. We were unable to extract information from a small number of Turkish, Polish, Hebrew, Italian, German and Chinese articles. Clinical or laboratory confirmation of dengue virus transmission found within these articles was entered into the database; any suspected cases were excluded. Reports of autochthonous (locally transmitted) cases or outbreaks were entered as an occurrence within the country in which transmission occurred. If imported cases were reported with information about the site of infection, they were geo-positioned to the country where transmission occurred. If imported cases were reported with no information about the site of contagion, they were not entered into the database. If an imported case led to autochthonous transmission within the recipient country and location information was available for the site of initial contagion and the site of the outbreak, this was recorded as two occurrences: one in the country of contagion and one in the country where the outbreak occurred.

Informal online data sources were collated automatically by the web-based system HealthMap (http://healthmap.org) as described elsewhere^4^. Briefly, HealthMap is an online infectious disease outbreak-monitoring system that captures data from a range of electronic sources in nine different languages. The system performs hourly scans of online news aggregators, listservs, electronic disease surveillance networks and public health outbreak report feeds. It captures four fields: headline (the headline, title or subject line), date (publication date), description (a brief summary) and information text (the main content of the article or report). The information text is passed to HealthMap’s classification engine, which parses out one or more disease names and outbreak locations using dictionaries of disease and location patterns. The system then uses a separate algorithm to assign relevance scores that classify alerts as (i) breaking (information about a new outbreak or new information about an on-going outbreak), (ii) context (content about research, policy or background on a particular disease), (iii) warning (articles that warn about the potential for an outbreak), (iv) not disease-related (articles that are captured by the system because they contain words that match disease names in the dictionary but are not in fact about an infectious disease) or (v) old news (an article that mentions a historical outbreak). Finally, HealthMap handles duplicates by aggregating together highly similar alerts such as those released by a news wire service and published in multiple periodicals. The requirements for including a dengue occurrence record from the HealthMap data set in our database were that the article or report contained the keywords ‘dengue’, ‘dengue fever’, ‘dengu’ or ‘dhf’ and was classified by the system as ‘breaking’. This HealthMap data set was last updated on 26th May 2012. Dengue reports since this time can be found on the dengue-specific HealthMap website (http://www.healthmap.org/dengue).

### Geo-positioning of data

All available location information was extracted from each peer-reviewed article and ProMED case report. The site name was used together with all contextual information provided about the site position to determine its latitudinal and longitudinal coordinates using Google Maps (https://www.maps.google.co.uk/). Place names are often duplicated within a country, so the contextual information was used to ensure the right site was selected. When the site name was not found, the contextual information was used to scan sites in the approximate area to check for names that had been transliterated in Google Maps in a different way to the published article (e.g., Imichli and Imishly). If the study site could be geo-positioned to a specific city, town or village, its centre was recorded and termed a ‘point location’ (i.e., associated with a specific latitude and longitude). Point occurrences also included explicit co-ordinates supplied in the article. In reports where more accurate details about the specific location within the city, town or village were available (such as a specific suburb), this was used to define the point occurrence. If the study site could only be identified at an administrative area level (e.g., province or district), its centroid of the polygon was recorded and overlaid later in a geographic information system (GIS) to record the appropriate administrative unit. All administrative units were as recognised by the FAO Global Administrative Unit Layer (GAUL) system^5^. These occurrences referring to an area were termed ‘polygon locations’. All formal occurrence records underwent spatial and temporal standardisation to ensure consistent definition of an occurrence before undergoing technical validation.

Geo-positions for the HealthMap data were generated using a custom-built gazetteer, or geographic dictionary, of over 4,000 relevant phrases and place names and their corresponding geographic coordinates. The system uses a look-up tree algorithm that searches for matches between sequences of words in alert info text and sequences of words in the gazetteer. When a match is found, a set of rules are applied which attempt to determine the relevance of the place name to the outbreak that is being reported based on the position of the phrase in the report text. The gazetteer includes place names at a range of spatial resolutions (e.g., neighbourhoods, cities, provinces and countries) and uses certain phrases to trigger exclusion of a place name (e.g., Brazil nut). All HealthMap records were added to the unstandardised database and then underwent spatial and temporal standardisation, as well as technical validation, along with the records from the literature and ProMED reports.

### Occurrence database management: locational and temporal standardisation

As the database was compiled from many different sources and by several persons, it was first necessary to standardise the data entries such that identical locations which may have been geo-positioned slightly differently were given the same unique identifier. As such, polygon records were all assigned a unique administrative code by overlaying the recorded geographic coordinates in the GIS with corresponding administrative unit shapefiles^5^. Point records were given the same unique identifier if they lay in the same 5 km×5 km pixel within a global grid. Finally, any record associated with a polygon measuring larger than 1°×1° at the equator was removed from the database, although the authors are happy to provide the records for these polygons upon request.

It was next necessary to temporally standardise the database, as the collected dengue occurrence data came in a variety of temporal forms. For example, some sources reported multiple cases in a single location throughout a year with no finer-scale temporal information. However, in other sources (particularly online sources), multiple cases in the same location throughout the year were presented as a new report each time subsequent transmission occurred. Furthermore, many sources described endemic transmission occurring across multiple years. As a result, we chose to define a single occurrence at a given unique location (as identified above) as one or more confirmed cases of dengue occurring within one calendar year, as this was the finest temporal resolution available across all records. This involved a procedure which: (i) disaggregated any records which were in the same location but spanning multiple years into individual records for each respective year; and then (ii) aggregated all records with the same unique location identifier and occurring within the same year to form a single occurrence record. The database in this form underwent technical validation before reaching its final state, as described below.

## Data Records

**Figure 1:**
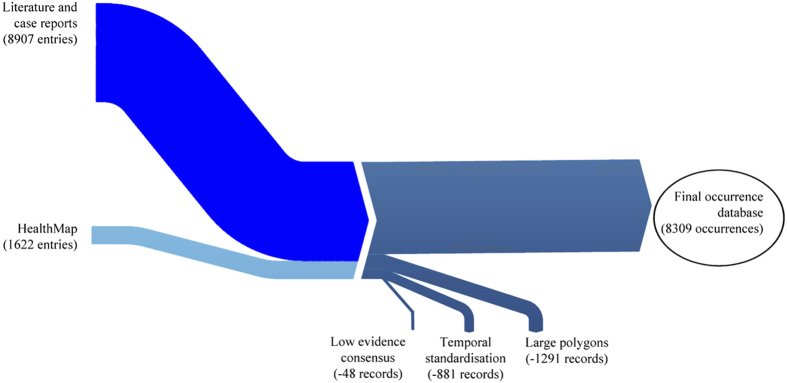
Occurrence data processing summary, beginning with the raw inputs and showing the proportion of data lost through the stages of quality control before reaching the final occurrence database.

**Figure 2:**
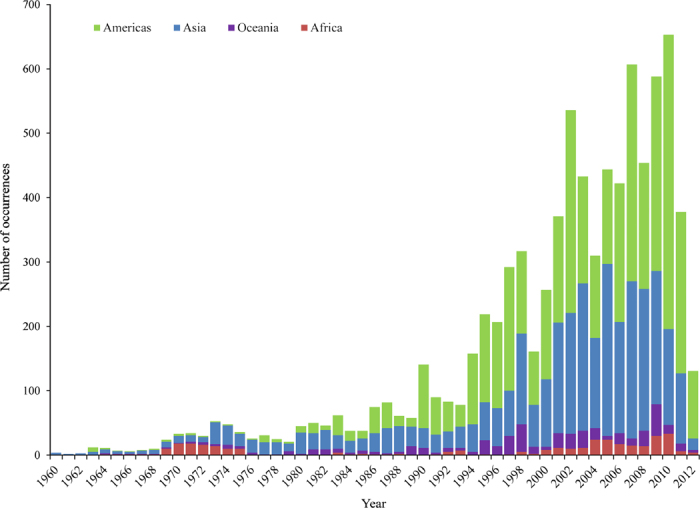
The numbers of occurrence locations per year separated by region.

The database is publicly available online (Data Citation 1) as a comma-delimited file for ease of use and the ability to import it into a variety of software programs. Each of the 8,309 rows represents a single occurrence record (one or more dengue cases in the same unique location within a single calendar year). A summary of the data management procedure, beginning with the raw inputs and showing the proportion of data lost through the stages of quality control before reaching the final occurrence database, is provided in Figure 1. Figure 2 displays the numbers of occurrence locations per year separated by region. The fields contained in the database are as follows:

1. **OCCURRENCE_ID:** Unique identifier for each occurrence in the database after temporal and locational standardisation.
2. **SOURCE_TYPE:** Manual entry versus HealthMap.
3. **LOCATION_TYPE:** Whether the record represents a point or a polygon location.
4. **ADMIN_LEVEL:** The administrative level which the record represents when the location type is a polygon. Values are 0 (national), 1 (state or province), 2 (district) and −999 when the location type is a point.
5. **GAUL_AD0:** The country-level global administrative unit layer (GAUL) code (see http://www.fao.org/geonetwork) which identifies the Admin-0 level occurrences as well as the Admin-0 polygon within which any smaller polygons and points lie.
6. **GAUL_AD1:** The first-level GAUL code which identifies the Admin-1 level occurrences as well as the Admin-1 polygon within which any smaller polygons and points lie. Values of −999 are assigned when the polygon was Admin-0 level.
7. **GAUL_AD2:** The second-level GAUL code which identifies the Admin-2 level occurrences as well as the Admin-2 polygon within which any points lie. Values of −999 are assigned when the polygon was Admin-0 or Admin-1 level.
8. **POINT_ID:** The unique identifier which was assigned to points falling within the same 5 km×5 km pixel.
9. **UNIQUE_LOCATION:** A unique identifier created for all locations (both points and polygons) based upon the point IDs and the GAUL codes.
10. **X:** The longitudinal coordinate of the point or polygon centroid (WGS1984 Datum).
11. **Y:** The latitudinal coordinate of the point or polygon centroid (WGS1984 Datum).
12. **YEAR:** The year of the occurrence.
13. **COUNTRY:** The name of the country within which the occurrence lies.
14. **REGION:** The region within which the occurrence lies – values are Asia, Oceania (includes Australia), Africa (includes the Arabian peninsula), and Americas.

## Technical Validation

The following procedures were carried out on the final database to ensure the accuracy and validity of the occurrence records.

1. A raster distinguishing land from water was created at a 5 km×5 km resolution and was used to ensure all disease occurrences were positioned on a valid land pixel.
2. We cross-validated all of the unique occurrence locations against dengue transmission extent based upon evidence consensus according to Brady *et al.*^6^ In brief, this classification was determined according to a qualitative evidence base that assessed consensus among a wide variety of evidence types on dengue presence or absence at a national and sometimes sub-national level. This consensus ranged from complete agreement on absence (score of −100) to complete agreement on presence (100). We chose to exclude points in areas with scores of less than −25. This conservative criterion was intended to preserve points in areas of both proven dengue presence and uncertainty on dengue status.
3. A random sub-sample of HealthMap occurrence points were manually checked to identify common geo-positioning problems which were rectified in the final database.

The result is a database consisting of 8,309 geo-positioned occurrences in total worldwide, broken down by region, location type and source type in Table 1. A map displaying their locations can be found in Supplementary Figure SA2 of Bhatt *et al.*^1^.

## Additional Information

**How to cite this article:** Messina, J. P. *et al.* A global compendium of human dengue virus occurrence. *Sci. Data* 1:140004 doi: 10.1038/sdata.2014.4 (2014).

## Supplementary Material



## Figures and Tables

**Table 1:** Occurrences broken down by region, location type and source type. All polygon information was manually entered from literature and case reports.

**Region**	**Points**		**Polygons**	**Total**
	**HealthMap**	**Literature & case reports**		
Africa	38	257	46	341
Americas	870	1934	1396	4200
Asia	324	1463	1502	3289
Oceania	27	303	149	479
***Total***	1259	3957	3093	8309
